# Behavioral Determinants of Routine Health Information System Data Use in Senegal: A Qualitative Inquiry Based on the Integrated Behavioral Model

**DOI:** 10.9745/GHSP-D-21-00686

**Published:** 2022-06-29

**Authors:** Pierre Muhoza, Haneefa Saleem, Adama Faye, Roger Tine, Abdoulaye Diaw, Almamy Malick Kante, Andrea Ruff, Melissa A. Marx

**Affiliations:** a Johns Hopkins Bloomberg School of Public Health, Baltimore, MD, USA.; b Institut de Santé et Développement, Université Cheikh Anta Diop de Dakar, Dakar, Senegal.; c Université Cheikh Anta Diop, Faculté de Médecine de Pharmacie et d'Odontologie, Dakar, Senegal.; d Direction de la Planification, de la Recherche et des Statistiques/Division du Système d'Information Sanitaire et Social, Ministère de la Santé et de l'Action Sociale, Dakar, Senegal.

## Abstract

Routine health information system (RHIS) data are essential in driving decision making and planning in health systems as well as health programs. However, despite their importance, these data are underutilized, and the underlying individual-level facilitators and barriers to use remain understudied. In this research, we applied the Integrated Behavior Model (IBM) to examine how attitudes toward RHIS data, perceived norms concerning RHIS data use, and the ability to use RHIS data influence the demand and use of RHIS data among stakeholders in Senegal. Using data from interviews with respondents working at national levels of malaria, HIV, and TB control programs in Senegal, we used a framework analysis approach to apply the IBM behavioral constructs and identify their linkages to RHIS data use. We found that attitudes about the quality, availability, and relevance of RHIS data for decision making were important in driving data use among respondents. Institutional expectations, organizational protocols, policies, and practices around RHIS data ultimately shape social norms around the use of the data. Although we found that perceived ability and self-efficacy to use RHIS data were not barriers to RHIS data use among stakeholders at the strategic levels of their respective organizations, these were reported to be barriers at lower levels of the health system. Low perceived control of the RHIS data production process ultimately reduced RHIS data use for decision making among the strategic-level respondents. We recommend context-specific reexamination of existing RHIS interventions with a renewed emphasis on behavioral aspects of data use. The IBM can help guide practitioners, policy makers, and academics to address multiple socioecological factors that influence data use behavior when recommending RHIS and data use solutions.

## Abstract (precis)

Although behavioral factors are thought to be important barriers to routine data use, they remain understudied particularly in low-income country settings. We show that the integrated behavior model can be a valuable theoretical framework for targeted communication strategies and capacity-building interventions aimed at promoting a culture of data use.

## Teaser

**Key Findings:**

- Respondents' instrumental and experiential attitudes about using routine health information system (RHIS) data for decision making revealed that they felt using RHIS data was time consuming, unreliable and that, often, the level of granularity was insufficient for their needs.
- Respondents' perceptions of lack of control over the data production, data sharing, and data dissemination processes affected their ability and comfort to use RHIS data consistently.
- Clear guidelines/protocols on routine data use, regular communications to health workers on issues related to routine data quality and use, and the organization of data review meetings are crucial for shaping a data use culture.

**Key Implications:**

- Policy makers need to develop a national plan solely dedicated to the improvement of routine data quality and use to address issues transcending disease-specific scopes.
- National stakeholders should redesign existing data use strengthening programs with a renewed emphasis on the behavioral aspects of routine data demand and data use.
- National stakeholders should consider approaches to strengthen pre-service training in data use competencies to complement the in-service capacity-building strategies.

Résumé en français à la fin de l'article.

## INTRODUCTION

Routine health information system (RHIS) data are crucial for informing public health policy, planning resource allocation, and guiding strategies for disease control programs. These data are broadly recognized as essential in driving planning and decision making at all levels of the health system.^1^^,^^2^ Around the globe, national governments and donors have invested considerable resources in an attempt to strengthen RHIS and produce actionable data.^3^^,^^4^ Despite the central role of RHIS in the health system and the investments made to improve RHIS data collection, multiple factors continue to impede the use of RHIS data in the decision making and strategy development processes. These factors include poor data quality,^5^^–^^7^ inadequate access to relevant data,^5^^,^^8^ insufficient data use competencies among potential users,^7^^–^^9^ as well as other organizational and health system-level determinants.^7^

The producers and users of RHIS data are individuals with interconnected professional roles within the health system.^10^ RHIS data use strengthening programs often focus on providing technical solutions and infrastructural support as well as building the capacity of both data users and producers.^11^ However, in many cases, capacity-building efforts often fail to address behavioral barriers to improved data use and production, resulting in meager and/or short-lived results. Behavioral barriers may be missed because they can be difficult to identify and address.^12^ According to the Performance of Routine System Management (PRISM) framework for designing and evaluating RHIS, behavioral influences on RHIS data use include intangible concepts such as the value that individuals ascribe to data, job responsibility, confidence levels for RHIS tasks, motivation to perform RHIS tasks and problem solving for RHIS tasks.^13^ Addressing these behavioral factors requires interventions that not only address competencies of RHIS data use but also influence attitudes, norms, and the broader culture on using RHIS data.^14^ Furthermore, the design of such interventions should be driven by behavior change frameworks that are informed by context-specific evidence on the behavioral determinants of data demand and data use.

The Integrated Behavior Model (IBM) is an adaptable theoretical framework that has been previously used to examine a variety of health behaviors^15^^–^^19^ and warrants further investigation in the context of data demand and use. It was originally conceptualized as a combination of leading health behavior research theories, such as the Theory of Reasoned Action and the Theory of Planned Behavior, thus making it more comprehensive.^18^^,^^20^^,^^21^ The IBM postulates that behavioral intention is the strongest determinant of human behavior, barring any situational barriers or deficiencies in skill or knowledge toward performing a given behavior. The model further posits that behavioral intention can be predicted by an individual's attitudes toward the behavior, perceived norms of the behavior, and one's perceived ability to perform the behavior.^15^^,^^20^^,^^22^ Attitudes arise from an assessment of the behavior itself (experiential attitudes), as well as beliefs about the likelihood that performing the behavior will have certain outcomes (instrumental attitudes). Norms are divided into: (1) injunctive norms, whether others in one's social group approve or disapprove of the behavior; and (2) descriptive norms, how common the behavior is within one's social group. Perceived ability is divided into perceived control and self-efficacy.^23^ Perceived control denotes the extent to which one feels responsible for their behavior. ^23^ Self-efficacy is a self-constructed concept relating to one's confidence in being able to perform a behavior despite the challenges that may arise.^19^ It should be noted that self-efficacy is a different concept from technical competence since one may have the requisite knowledge and skills to perform a behavior while simultaneously lacking the confidence or sense of agency to perform the behavior. Furthermore, an individual may be unaware of the gap between their perceived and actual competence to perform a behavior, potentially creating opportunities for specific interventions to bridge the gap to meet the expected behavior.^24^^,^^25^

The Integrated Behavior Model is an adaptable theoretical framework that warrants further investigation in the context of data demand and use.

This study aimed to examine how attitudes toward RHIS data, perceived norms concerning RHIS data use, and the ability to use RHIS data influence the actual use of the data to drive programmatic processes in Senegal by applying the IBM. By examining how these constructs influence RHIS data use, one can learn whether certain individual-level factors are more salient determinants of RHIS data use to specific cadres of users. Determining whether RHIS data use is more attitudinally driven than normatively driven, for instance, may help inform the design of future messaging interventions targeting specific cadres of data users in Senegal. Thus, this work may potentially inform future strategies by the Senegal Ministry of Health (MOH) to improve the quality and use of local RHIS data, an important policy priority as the MOH and its partners continue to strengthen the District Health Information Software version 2 (DHIS2), Senegal's national RHIS.^26^^–^^29^

## METHODS

This qualitative study was conducted between January 2019 and November 2019 as part of a broader qualitative research project aiming to identify the determinants of RHIS data use in Senegal.^27^ In this study, we treat data demand and data use as modifiable health behaviors. The study includes the first 2 steps outlined by Yzer^19^ for determining effective, targeted health communications: (1) clear definition of the health behavior to be targeted, and (2) gathering information directly from the population to be targeted with the behavior change effort (Box).

BOX **Operational Definitions for Qualitative Study of RHIS Data Use**

For the purposes of this study, we use the following definitions.

**RHIS data production**: RHIS management and data collection at the community or facility level for dissemination to subnational, and eventually, national levels.

**Data use^a^**: The review or analysis of routine data to inform decision making.

**Data demand^a^**: As recommended by MEASURE Evaluation, the valuing of routine data or the active request of routine data for the purposes of using it for decision making.^12^ We further equate the act of data demand with the intention of using the data.

**Decision making**: The explicit consideration of information by stakeholders in the process of policy making, program planning and management, or service provision, even if the final decision or actions are not based on that information.

Abbreviation: RHIS, routine health information system.

^a^As others have noted, it may be difficult, in practice, to distinguish between data demand and data use.^12^ Therefore, in this study, we treat them as parts of a single process.^12^

### Data and Data Analysis

Details on the study design, setting, participants as well as the steps taken to ensure quality have been described elsewhere.^27^ Briefly, data come from in-depth interviews conducted with 18 key informants of which 9 were high-level decision makers within their organizations and 9 were mid-level personnel. The key informants were purposively selected based on their affiliation with organizations active in the malaria, TB, and HIV programmatic areas in Senegal. Ultimately, decision makers included program directors, program coordinators, program managers, and program officers from organizations ranging from divisions of the MOH, civil society, and implementing partner organizations with local or international scopes. The mid-level personnel category included monitoring and evaluation officers and data analysts employed in the aforementioned organizations. All respondents were employed in organizations that were current or past recipients of programmatic funding for HIV, TB, or malaria activities awarded directly or indirectly by a single donor organization. The broader parent project of this qualitative research study aimed to understand national-level data use processes in Senegal. Consequently, the sampling frame of the respondents was restricted to individuals working at the central level of the health system or their respective organizations.

The informants were asked questions related to the decision-making processes and information flow in their organization, their attitudes toward routine data and their quality, the barriers and facilitators influencing the use of routine data, their perceptions of the existing best practices as they relate to routine data and recommendations to improve the use of routine data. Questions were adapted from the MEASURE Evaluation tool to develop a semistructured interview guide that allowed an assessment of RHIS data demand and constraints to data use.^30^ The adapted guide is provided in the Supplement. Interviews took place in person in the private offices of the respondents. They were conducted in French and took between 30 and 45 minutes. The interviews were audio-recorded, transcribed verbatim, and proofread by a researcher with native fluency in French and graduate training in qualitative research methods.

We employed a framework analysis approach using 5 steps: (1) familiarization of interview transcript content, (2) identification of thematic frameworks, (3) indexing, (4) charting, and (5) interpretation.^31^ Interview transcripts were analyzed using a concept-driven (deductive) coding approach that was informed by concepts drawn from the IBM. The coding framework included 4 primary categories: attitudes (with subcodes for experiential and instrumental attitudes), perceived norms (with subcodes for descriptive and injunctive norms), perceived ability (with subcodes for perceived control and self-efficacy) as well as knowledge and skills. Given that the major situational constraints to data use such as the sociopolitical, organizational, and system design factors influencing RHIS data use in Senegal have been discussed in previous research,^27^ this study placed greater emphasis on the other aspects of the IBM. Analyses were conducted using Atlas.ti 7.5 and Microsoft Excel. Results are presented as direct quotes from interviews and serve as supporting evidence for the themes that emerged from the analyses.

### Ensuring Quality and Rigor

After the initial analysis of the collected data, we summarized the emergent themes and shared them with the key informants for respondent validation. This was done to verify the credibility and validity of our research findings. The emergent themes were sent to respondents via an online survey (Qualtrics). Respondents were invited to reflect critically on the findings and offer input on any instances of misinterpretation of facts. During the sharing of emergent themes, respondents were also invited to provide additional recommendations to improve data use. Respondent input during the respondent validation procedure was treated as additional data.

### Ethical Approval

The protocol for this study was reviewed and approved by 3 ethics committees: the Institutional Review Boards at the Johns Hopkins University, Université Cheikh Anta Diop de Dakar, and the National Ethics Committee for Health Research in Senegal (reference number: 00000015/MSAS/DPRS/CNERS). Before each interview, we explained the study objectives, risks, and benefits to study participants and answered any questions they had. Each study participant gave their informed written consent.

## RESULTS

We depict the relationships between the different IBM constructs within the context of RHIS data use (Figure). RHIS data use is primarily determined by data demand (i.e., intention to use RHIS data) but also by competency to use RHIS data (i.e., knowledge and skills for RHIS data use) and situational constraints affecting RHIS data use processes. Data demand is affected by several factors, including attitudes about the quality, availability, and relevance of RHIS data for decision making, as well as perceived norms around RHIS data use and an individual's perceived ability to use RHIS data. We present the emergent themes from the Senegal context serving as supporting evidence for the different constructs in the model and in the subsequent sections.

**FIGURE:**
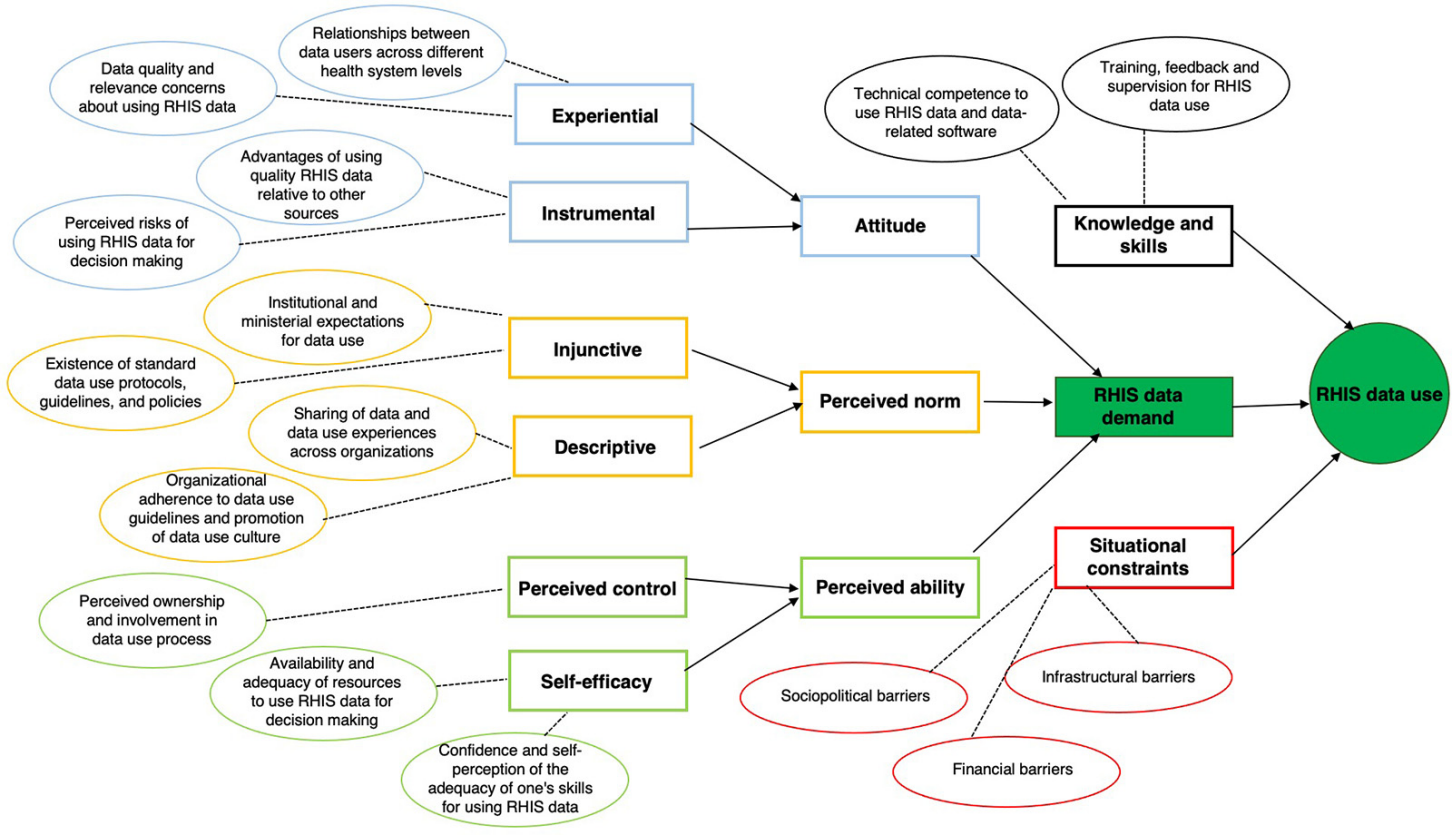
IBM for RHIS Data Use^a^ Abbreviations: IBM, integrated behavior model; RHIS, routine health information system. ^a^Rectangles represent constructs adapted from Fishbein et al.'s IBM.^22^ Ovals represent emergent themes relevant for RHIS data use. This model assumes that data demand is the intention to use RHIS data and that RHIS data demand and data use are parts of a single process.

RHIS data use is determined by data demand, competency to use RHIS data, and situational constraints affecting RHIS data use processes.

### Attitudes

Compared to the other constructs within the IBM, we found that attitudes about RHIS data, the quality of RHIS data, and the outcomes of using quality RHIS data were the most commonly reported drivers of using the data. Respondents' experiential attitudes were often the beliefs attached to or feelings toward using RHIS data relative to its quality. In this regard, several respondents felt that using RHIS data was often a stressful and time-consuming task given the amount of effort required to gather often incomplete data from different sources on short deadlines, summarize them into actionable information, and disseminate them where they are needed.

*Working with routine data is excessively tedious. It wears you down because you run around trying to make sure that the data are complete. Often, you are forced to fight to get the data on time and be able to analyze them. Sometimes you don't even have time to think about the analysis because by the time the data comes, the report is already due. Sometimes you just have to make do with what you have. I think it is an endless struggle. At each reporting period, it is an effort to receive, analyze and process the data.* —Mid-level personnel #1

Other commonly cited experiential attitudes that were noted included attitudes related to the relevance of RHIS data for decision making. In this regard, the granularity of RHIS data emerged as a particularly important determinant of RHIS data demand and thus data use. We found many instances in which informants perceived existing RHIS data to be insufficiently granular for their information needs.

*The problem with the DHIS2 is the level of detail in the system. The DHIS2 is not easy to use because the data we are provided with is aggregated at district and national levels whereas we need data from the communities in which we work.* —Mid-level personnel #7

In fact, these perceptions affected the value that users place on the RHIS data and why they preferred to use alternative data sources (e.g., surveys) or establish parallel data collection systems. In contrast, instrumental attitudes were often related to the importance of using RHIS data to guide decision making as well as the perceived potential consequences of using routine data of poor quality.

*If you don't use quality routine data, it could lead to the failure of the entire project. All the decisions will be based on the wrong information. Hence the importance of having very good quality data. And we really do make sure that we have quality information that will allow us to make the right decisions without the risk of errors.* —Mid-level personnel #3

Instrumental attitudes were often related to the importance of using RHIS data to guide decision making as well as the perceived potential consequences of using routine data of poor quality.

Furthermore, instrumental attitudes about RHIS data and its quality in Senegal appeared to be important drivers of whether the data were used or whether alternative sources of data were perceived to be more reliable. A key driver of the development of these particular attitudes was the belief that RHIS data are not consistently accessible or available when needed for use. Retention of RHIS data during health worker strikes and unclear data sharing policies that limit RHIS access for certain stakeholders contributed to this perception.

We found a general belief among respondents that individuals in influential positions at lower levels of the health system rarely used routine data for decision making due to prevailing negative experiential attitudes. National-level respondents generally expressed low trust in the data use competencies of data producers and users in the lower levels of the health system/organizations as well as their level of engagement and understanding of the value of data. They believed that to achieve high levels of routine data use within the broader health system, attitudes among data users at the operational level of the health system have to be addressed.

*[For interventions] to be effective, data have to be valued at all levels. And data-driven decisions have to be made at all levels. People have to understand that data are not just for me to send to the Ministry, but it is information that I have to first check and use. These are data that can also allow me to guide my own strategies.* —Decision maker #1

Several respondents saw the bottom-up approach of reinforcing positive instrumental attitudes at lower levels of the system as important in promoting a culture of accountability, as well as in valuing and using routine data. Suggested opportunities to reinforce the attitudes included during periodic workforce training activities as well as during pre-service training.

*I don't think we have a good data culture yet. I think we would gain tremendously by emphasizing the importance of data during our staff's pedagogical training. In academic training schools, whether it be at university or whether it be in national midwifery nursing schools or in other health institutions.* —Decision maker #2

### Social Norms

Social norms were also important determinants of the demand and use of RHIS data. Respondents from organizations within the MOH often reported that the establishment of guidelines, standard operating procedures, and manuals improved the use of RHIS data by setting norms and guidelines around data use.

*It is true that we must train people and all that. But I also think that it was fundamental that at some point we introduced a procedural manual. I believe it is important when people have a document that they can always refer to for a clear description of data management procedures and what is expected.* —Decision maker #7

Social norms were also important determinants of the demand and use of RHIS data.

Furthermore, some stakeholders pointed out that the availability of bulletins and newsletters enabled the comparison of performance indicators across districts and set standard expectations.

*Recently, I have noticed that the Directorate of Planning, Research and Statistics produces a quarterly electronic newsletter on data management… that they disseminate in the medical officer network in the regions. So, this allows people to have information on [data] completeness by region and make some comparisons. And that too can elicit some reactions and feedback…Sometimes they even do it by district and share it across the network. And that actually leads to an overall gradual improvement in monitoring.* —Decision maker #4

Multiple respondents pointed out that many data users, particularly region-level and district-level decision makers, valued the opinion of their peers from other parts of the country and thus strived to produce better quality data and use data more effectively. Respondents recounted that they had the chance to observe the performance of peers during regularly disseminated bulletins and quarterly data review meetings.

*The organization of data review meetings is a good practice…because we get to examine and correct the data together. Whereas [the meetings] were initially focused on data collection, the focus has gradually shifted towards deeper data analyses. And I think this is a good practice that not only improves data collection, but also makes the districts…more aware of the importance of routine data and the need for quality data. Because these are big meetings and each district representative presents their data and then people provide positive or negative criticism. So, people are more inclined to pay attention to the data they produce and use.* —Decision maker #7

### Perceived Ability

Within the construct of perceived ability, we found perceived control of RHIS data use to be much more salient to study participants compared to their reported self-efficacy in using the data.

In general, there was a feeling among respondents that they lacked control over the data production, data sharing, and data dissemination processes that in turn affected users' beliefs in their ability and comfort to use RHIS data consistently. In essence, the availability and quality of the RHIS data appeared to depend more on the capacities and engagement of data producers. In this regard, the recurrent health data retention strikes in Senegal emerged as an important factor that reduced RHIS data availability and respondents' comfort with relying on the data to inform decision making.

*When your decisions are based on routine data, you are confronted with external factors such as strikes or data retention. So to make the commitment that my report will be based on data that I don't produce, that's a big deal. It's a big challenge. Because if there is a data retention strike today, there will be indicators that we can't report on. And that's because we don't produce the information.* —Decision maker #4

Respondents felt that they lacked control over the data production, data sharing, and data dissemination processes that in turn affected users' beliefs in their ability and comfort to use RHIS data consistently.

Among civil society respondents, the lack of direct access to national RHIS data and lack of clarity on data sharing policies were reported to hinder their ability to review RHIS data and to assess their accuracy. This lack of control of data collection affected both their level of trust in the data as well as their sense of involvement in the national processes related to RHIS data.

*It's not easy to get [DHIS2] data. Up to this point we are still trying… Because all we want is direct access to the system. This would allow us to see all the activities in a region, progress on indicators in a region. This would also allow us to reorient certain things. We once had access to the system through an external person… we noticed that [where we work], information and many activities from [our organization] had not been added. So we drew attention to this problem… If we had this access, it would allow us to do these checks.* —Mid-level personnel #3

Lacking confidence in the data use competencies that are required to use RHIS data did not appear to be a barrier to using routine data among the study participants themselves. Nonetheless, respondents repeatedly asserted that these were important among the data users working at the lower levels of the health system or in some cases, their respective organizations. Consensus emerged that the perceived low levels of skills for data use, lack of necessary equipment, lack of office space, and inadequate wages contribute to the low motivation among health workers to use RHIS data.

*Some of these analyses can be done at the regional level. But if there are no resources and support available in the health system, people are not motivated. Yet, all the technical know-how exists at the medical region and health district levels… But unfortunately, not everyone has the same commitment. As you also know, recently there have been strikes among the health workers, meaning that their demands are not being met. So, all this contributes to the demotivation of health workers to work on the data.* — Decision maker #6

### Skills and Knowledge Related to Data Use

Similar to the observation that the self-efficacy to use RHIS data was not noted as a salient construct among study participants themselves, we also failed to find evidence pointing to the lack of technical skills as a barrier to the use of RHIS data among the interviewed high- and mid-level personnel. Nonetheless, it was clear that study respondents valued technically competent data managers who are able to fully exploit RHIS data and draw actionable information from them.

*It would be good to strengthen the overall capacity of everyone involved in data processing and data management. Because there are a lot of people who are in those positions who don't truly understand data and its importance. Or how to make people learn more from data. So, there is a need for further capacity building of all those who are involved in the data chain.* —Mid-level personnel #8

Respondents pointed to the need to strengthen the capacity of all data users throughout the health system and at all levels of their organizations. They referenced the reinforcement of existing interventions such as training workshops, coaching, and supervision as means of strengthening staff capacity.

*I think we need to strengthen the monitoring and evaluation teams. Data managers need to be adequately trained, empowered and supported.* —Decision maker #2

## DISCUSSION

Using the IBM as a theoretical framework to understand RHIS data use behavior in the Senegal context, we found qualitative evidence suggesting that attitudes about the quality, availability, and relevance of RHIS data for decision making, as well as perceptions of the outcomes of using quality data, were the dominant drivers of RHIS data use. Both positive and negative experiential attitudes were common across all respondents. In particular, respondents broadly acknowledged the importance of RHIS data and the role of their quality in guiding the decision-making process. These experiential attitudes appeared to be common knowledge within this population suggesting that they would not be important targets for behavior change communication among data users working at strategic levels of their organizations. For this cadre of data user, it may be far more effective to target the instrumental attitudes rooted in the beliefs that RHIS data are not reliable for guiding decision making due to their poor quality and inconsistent availability.

Of note, findings highlighted the concept of trust as an overarching theme underpinning various respondent concerns. In particular, the mistrust of RHIS data as a true reflection of realities in the field, the mistrust of the RHIS capacities and engagement of staff working in the lower levels of the health system, and the mistrust of the reliable availability of RHIS data were key concerns. Potential interventions that could be employed include communications of success stories and lessons learned in matters related to RHIS data use together with the promotion of role modeling from the leading health authorities on matters related to the use of RHIS data, notwithstanding the data quality concerns.^26^^,^^32^ With regular demonstrations that RHIS data can be successfully used to guide programs, decision makers may gradually begin to trust the data.

Policy makers should explore solutions to some of the systemic problems that influence the availability and accessibility of RHIS data in Senegal. For example, articulating clear policies on database access and data sharing may promote transparency and allay some of the concerns that civil society actors have regarding using RHIS data. Based on the perceived lack of involvement in data production and dissemination processes in Senegal found within this population, it would be important to include the perspectives of civil society actors in policy development. On a broader scale, Senegal needs to develop a national strategic plan solely dedicated to the improvement of RHIS data quality and use. Although the current approach of having an RHIS module in program-specific plans is laudable, a framework focused on RHIS can enable the country to set priorities that transcend individual programs. Furthermore, such a framework would be an opportunity for health authorities to articulate plans for sustainable financing of RHIS and address the issues underlying data retention strikes and irregular district-level data quality reviews. We have previously discussed additional approaches that have the potential to help address these latter issues.^27^

Policy makers should explore solutions to some of the systemic problems that influence the availability and accessibility of RHIS data in Senegal.

On a broader scale, Senegal needs to develop a national strategic plan solely dedicated to the improvement of RHIS data quality and use.

Besides the development of policies and strategic frameworks, we note that elements of some of the interventions capable of addressing the identified data user attitudes are already in place. For instance, the regularly disseminated bulletins and newsletters that document trends in indicators of data quality and positive data use experiences were often cited by respondents as some of the best practices that help them stay abreast of developments and encourage them to use RHIS data. Furthermore, the quarterly data review meetings that bring together actors from all levels of the health system are an additional avenue to affect individual data user attitudes on a broad scale. As Braa et al. demonstrated in their evaluation of workshops focused on improving RHIS data quality and use among Tanzanian stakeholders, these types of regular workshops can stimulate gradual changes in mindsets and practices by encouraging RHIS data use in small incremental steps.^33^ Their study also showed that the workshops allow data users to progressively improve their problem-solving skills and confidence in performing RHIS tasks thereby addressing the IBM constructs of skills and self-efficacy, respectively.^33^

The advantages of exploiting these preexisting mechanisms extend beyond merely changing attitudes and engaging data users at the individual level. At the ecological level, they also contribute to the establishment of norms around data use (both descriptive and injunctive) potentially promoting progress towards a shared data use culture. This may happen through personalized normative feedback, where people receive information on how they are performing against others around them thereby motivating them towards performing the desired behavior.^34^^,^^35^ Importantly, they facilitate teamwork and enable actors to feel involved in the broader RHIS improvement efforts.

Based on the consensus among respondents and on evidence from literature^36^^,^^37^ that data use issues are more pronounced at facility and district levels of the health system compared to national levels, future studies should focus on further clarifying the behavioral determinants at that level. This understanding can help inform the design of interventions and the setting of priorities to address any identified challenges. In particular, competencies and personal agency for RHIS tasks were reported to be areas of concern among decision makers and data managers working at the operational levels of the health system. This observation suggested the need for developing specific strategies to stimulate the use of RHIS data as well as strengthening the existing ones. Coaching, supervision, and the provision of regular feedback to staff are some of the mechanisms that were cited as existing best practices in RHIS data use. Others have previously shown that they improve staff knowledge, skills, and self-efficacy to perform RHIS tasks.^38^^–^^40^ Additionally, experience elsewhere in Africa suggests that supportive job aids for RHIS tasks should be a critical component of data use capacity-building frameworks for health care workers at the point of care.^41^ The suitability of these tools in the Senegal context should be explored. Finally, national stakeholders should also consider approaches to strengthen pre-service training in data use competencies to complement the in-service capacity-building strategies. As highlighted by respondents in this study and discussed elsewhere, this may include reviewing training curricula in health training institutions to emphasize RHIS data use competencies.^42^ It may also include retraining tutors on current trends in RHIS data use and tools. Resources have been developed that may assist in these efforts.^43^

Finally, national stakeholders should also consider approaches to strengthen pre-service training in data use competencies to complement the in-service capacity-building strategies.

Given that the availability of granular data also emerged as an important determinant of RHIS data use, ensuring access to detailed data is critical to maximize its use. Several respondents found data aggregated at district and national levels irrelevant for their needs despite RHIS data being typically captured at the individual level and summarized by health facility. Thus, it is essential to revisit data-sharing practices and policies in Senegal to ensure that end users have granular data that are relevant to their needs. Strengthening the implementation of the DHIS2 tracker module to facilitate the collection and dissemination of individual-level data should also be a priority.^44^^,^^45^

Understanding the behavioral determinants of RHIS data use is a crucial yet seldom addressed topic in the literature, particularly in the context of low- and middle-income countries.^14^ The few studies that have addressed this issue made no attempt to organize the behavioral determinants into a behavior change model that can be used to improve RHIS processes and performance.^46^^–^^49^ Ohers have sought to evaluate the organizational and behavioral factors through the PRISM framework in low- and middle-income country settings.^32^ Although the value of the PRISM framework is well established, the approach is limited in some respects regarding the behavioral influences it directly addresses. For instance, unlike the IBM we describe here, the PRISM framework does not directly address attitudes toward RHIS data and data quality, which we found to be frequently cited determinants of data use in the Senegal context. Given the need for an approach that can comprehensively and systematically integrate behavioral perspectives in broader RHIS interventions, we propose that future studies evaluate the potential of the IBM to inform RHIS data use interventions.

### Strengths and Limitations

An important strength of this study was the incorporation of concepts from current practice and contextual realities into a theoretical health behavior model. Grounding theoretical behavior change interventions in practice may increase their accessibility to practitioners.^50^ In addition to enabling the identification of barriers that may be targeted for the improvement of RHIS data use in Senegal, our analysis also allowed us to both identify current best practices as well as suggest modifications to existing practices that are socially and culturally compatible with the local context.

Our analysis also allowed us to both identify current best practices as well as suggest modifications to existing practices that are socially and culturally compatible with the local context.

Nevertheless, this study was also bound by some limitations. Qualitative data collection and analysis are not objective processes; the researcher can either disregard or attend to observations and information.^51^ Although we attempted to assemble a diverse group of key informants in our purposive sampling strategy, the inherent limitation of purposive recruitment is the restriction on the transferability of findings to other contexts or cadres of RHIS data users.^52^ Furthermore, the experiences of the study participants may not be completely transferrable to other health professionals in Senegal nor to other professionals in different countries. The former is important to remember while interpreting study findings, given the fact that study participants were employed at the national level whereas they often alluded to issues at the district or facility levels of the health system. Due to limited study resources, we were unable to include the perspectives of key informants from these lower levels of the health system. Although this may limit our ability to make definitive conclusions about the behavioral determinants of RHIS data use at those levels, the high degree of agreement among the different respondents suggests that our findings may be reliable. Nonetheless, future studies should apply the IBM for RHIS data use at the operational levels of the health system to provide a more comprehensive understanding of the behavioral determinants influencing data use in Senegal.

We are unable to comment definitively to what extent the individual constructs of the IBM contribute to the determination of the intention to use data. As Cislaghi and Heise have cautioned, the prevalence of a norm (or an attitude) does not necessarily indicate its strength.^53^ Although the recurrent nature of determinants addressed by the IBM may suggest that these determinants are the most likely to influence the use of RHIS data, additional studies with larger sample sizes supplemented with quantitative methods are needed to augment our understanding of the relative contributions of individual constructs and the correlations among them. The quantitative methods may include structural equation modeling approaches such as those previously employed by others to quantify the impact of individual factors on the intention to perform other behaviors examined in the context of the IBM.^54^^–^^56^

Finally, we are also unable to determine conclusively the comparative effect of barriers versus facilitators on the use of RHIS data. Admittedly, based on the frequency of recurrence of behavioral determinants in our analysis, barriers were more recurrent compared to facilitators. As discussed earlier, however, the frequency of a factor does not equal its importance to the performance of the behavior. It is possible that indeed the higher frequency of barriers may be suggestive of their perceived importance to the study participants, as well as their peers, or it may suggest that barriers were easier for participants to articulate in the context of an interview. Future studies should further investigate the relative impact of barriers versus facilitators on RHIS data use.

## CONCLUSION

Sustaining the gains resulting from RHIS data use strengthening programs ultimately depends on the ability to promote a culture where data are not only valued at the individual, organizational, and policy levels but also used consistently to drive action. Achieving this objective in the Senegal context will require targeted messaging interventions to address the prevailing perceptions among data users that RHIS data are of poor quality, not reliably available for use, and not relevant for decision-making needs. To be effective, the messaging interventions will need to be tailored for the different levels of end users (national, district, and facility levels). Developing more robust policies that improve RHIS data access and sharing among the different stakeholders in Senegal would also send a strong message of MOH support for a sustained data use culture. Furthermore, the IBM for data use may also serve as an effective framework to improve upon the existing interventions such as the data review meetings, data dissemination mechanisms, supportive supervision, and the various capacity-building initiatives by ensuring that multiple levels of behavioral influence are addressed in intervention design. Additional studies are needed to quantify the relative importance of the specific IBM constructs, as well as to clarify the comparative influence of identified barriers and facilitators on RHIS data use. Finally, the present study underscores the need to explore the behavioral determinants of RHIS data demand and use at facility and district levels for a more comprehensive understanding of priority areas for action at all levels of the Senegalese health system.

## Supplementary Material

GHSP-D-21-00686-supplement.pdf

## Translation

**En Français**

**Déterminants comportementaux de l'utilisation des données du système d'information sanitaire de routine au Sénégal: Une enquête qualitative basée sur le modèle intégré du comportement**

**Résumé**

Les données du système d'information sanitaire de routine (SISR) sont essentielles pour guider la prise de décision et la planification des systèmes de santé et des programmes de santé. Cependant, leur utilisation reste faible. Les facilitateurs et les obstacles à leur utilisation au niveau individuel restent peu étudiés. Dans cette recherche, nous avons utilisé le modèle intégré du comportement (MIC) pour examiner comment les attitudes envers les données du SISR, les normes perçues concernant leur utilisation et la capacité à les utiliser influencent-elles leur demande et leur utilisation parmi les parties prenantes au Sénégal. En nous basant sur des entretiens avec des personnes travaillant au niveau national dans les programmes de lutte contre le paludisme, le VIH/Sida et la tuberculose au Sénégal, nous avons utilisé une approche d'analyse de cadre pour appliquer les concepts comportementaux du MIC et identifier leurs liens avec l'utilisation des données du SISR. Les attitudes concernant la qualité, la disponibilité et la pertinence des données du SISR pour la prise de décision étaient importantes à leur utilisation parmi les répondants. Les attentes institutionnelles, les protocoles organisationnels, les politiques et les pratiques concernant les données du SISR déterminent finalement les normes sociales relatives à leur utilisation. Bien que nous ayons constaté que la perception de la capacité et l'auto-efficacité dans l'utilisation des données du SISR ne constituaient pas des obstacles à leur utilisation parmi les parties prenantes au niveau central de leurs organisations respectives, ces facteurs ont été mentionnés comme des obstacles importants aux niveaux inférieurs du système de santé. La perception d'un faible contrôle du processus de production des données du SISR réduit finalement leur utilisation pour la prise de décision parmi les répondants du niveau central. Nous recommandons un réexamen contextuel des interventions existantes en matière de SISR en mettant l'accent sur les aspects comportementaux de l'utilisation des données. Le MIC peut aider à guider les praticiens, les décideurs et les chercheurs à prendre en compte les multiples facteurs socio-écologiques qui influencent les comportements d'utilisation des données du SISR lorsqu'ils recommandent des solutions relatives aux SISRs et à l'utilisation de leurs données.

**Peer Reviewed**

**First published online:** May 25, 2022.
