# Supplementary material for: Behavioral Determinants of Routine Health Information System Data Use in Senegal: A Qualitative Inquiry Based on the Integrated Behavioral Model
Source: Glob Health Sci Pract. 2022 Jun 29;10(3):e2100686. doi: 10.9745/GHSP-D-21-00686 (PMC9242607; doi:10.9745/GHSP-D-21-00686)
Supplement: GHSP-D-21-00686-supplement.pdf [file GHSP-D-21-00686-supplement.pdf]

Supplement to: Muhoza P, Saleem H, Faye A, et al. Behavioral determinants of routine health information system data use in Senegal: a qualitative inquiry based on the integrated behavioral model. *Glob Health Sci Pract*. 2022;10(3):e2100686. https://doi.org/10.9745/GHSP-D-21-00686

In-depth interview guide

Priority questions for high-level decision-makers

1. In general, what is the decision-making process like in your organization?

Types of data used in professional capacity

2. In general, what are the main considerations you make when making a decision about your program's direction or setting priorities?
    a. (If data is not mentioned) How important is data in this decision-making process?
    b. Where do you get information on how your programs are doing?

Barriers and facilitators to data use

3. What specific challenges have you experienced personally or among your staff when it comes to using routine data?
4. What are some of the factors would you say have improved or have the potential to improve your ability to use routine data specifically?
5. Are there some factors beyond your control that affect your ability to use routine data in your work?
6. In what ways would you say your organization influences employees who should be using routine data in their work to actually use it?
7. What are some of the challenges you experience in sharing routine data across organizations and agencies?

Attitudes towards data quality

8. How do you think the quality of routine data affects your use of routine data?

Existing best practices in data use

9. Now I want to ask about some of the currently existing practices you may have observed in your work that improve your ability or the staff's ability to use routine data. To get a good sense of these practices, let us separate the practices by the different phases that routine data goes through. What are some good practices you have seen:
    i. At the data collection level?
    ii. At the data review, management and analysis stage?
    iii. At the data interpretation and dissemination stage?

Recommendations to improve data use

10. What are some of the changes you would like to see implemented to enable you to use routine data better or make the data more useful for your job?
11. What type of resources would you like to receive to improve your ability to use routine data in your organization?

Supplement to: Muhoza P, Saleem H, Faye A, et al. Behavioral determinants of routine health information system data use in Senegal: a qualitative inquiry based on the integrated behavioral model. *Glob Health Sci Pract*. 2022;10(3):e2100686. https://doi.org/10.9745/GHSP-D-21-00686

Priority questions for National-Level Mid-Level Analysts and M&E Personnel

Types of data used in professional capacity

1. In general, what are the main considerations your organization makes when making a decision about the program's direction or setting priorities?
    a. (If data is not mentioned) How important is data in this decision-making process?
    b. Where do you get information on how your programs are doing?

Attitudes towards data quality

2. How do you think the quality of routine data affects your use of routine data?
3. How do you decide if the data that you report/receive are good quality?

Barriers and facilitators to data use

4. What specific challenges have you experienced personally or among your peers when it comes to using routine data?
5. What are some of the factors would you say have improved or have the potential to improve your ability to use routine data specifically?
6. Are there some factors beyond your control that affect your ability to use routine data?
7. In what ways would you say your organization influences employees who should be using routine data in their work to actually use it?

Existing best practices in data use

8. Now I want to ask about some of the currently existing practices you may have observed in your work that improve your ability or the staff's ability to use routine data. To get a good sense of these practices, let us separate the practices by the different phases that routine data goes through. What are some good practices you have seen:
    i. At the data collection level?
    ii. At the data review, management and analysis stage?
    iii. At the data interpretation and dissemination stage?

Recommendations to improve data use

9. What are some of the changes you would like to see implemented to enable you to use routine data better or make the data more useful for your job?
10. What type of resources would you like to receive to improve your ability to use routine data?
11. Are there changes you could make to the process to improve the efficiency of using data?
12. As we conclude, are there any other thoughts that you would like to share on data, data collection, reporting, or quality?
